# [Retracted] β-catenin signaling induces the osteoblastogenic differentiation of human pre-osteoblastic and bone marrow stromal cells mainly through the upregulation of osterix expression

**DOI:** 10.3892/ijmm.2026.5917

**Published:** 2026-07-06

**Authors:** Bo Liu, Song Wu, Lihua Han, Chaoyue Zhang

Int J Mol Med 36: 1572-1582, 2015; DOI: 10.3892/ijmm.2015.2382

Following the publication of this paper, it was drawn to the Editor's attention by a concerned reader that certain of the western blot data featured in Figs. 1A and 5A were strikingly similar to data that had already been submitted to the journal *Oncology Reports* in an article written by different authors; in addition, some of the individual protein blots in Fig. 1A of the above paper were remarkably similar to blots that appeared in Fig. 2A of the paper submitted to *Oncology Reports*, suggesting that there may have been some inapproprate editing of the bands in question. Finally, upon assessing the data in this paper independently in the Editorial Office, it came to light that control western blot data in Fig. 1A were also strikingly similar to data in another paper submitted for publication to the journal *PLoS One*, which again had been written by different authors.

Owing to the fact that the contentious western blot data in the above article were found to be strikingly similar to data that had already been submitted for publication elsewhere, the Editor of *International Journal of Molecular Medicine* has decided that this paper should be retracted from the Journal. The authors were asked for an explanation to account for these concerns, but the Editorial Office did not receive a reply. The Editor apologizes to the readership for any inconvenience caused.

